# HLA-Driven Convergence of HIV-1 Viral Subtypes B and F Toward the Adaptation to Immune Responses in Human Populations

**DOI:** 10.1371/journal.pone.0003429

**Published:** 2008-10-21

**Authors:** Dario Alberto Dilernia, Leandro Jones, Sabrina Rodriguez, Gabriela Turk, Andrea E. Rubio, Sandra Pampuro, Manuel Gomez-Carrillo, Christian Bautista, Gabriel Deluchi, Jorge Benetucci, María Beatriz Lasala, Leonardo Lourtau, Marcelo Horacio Losso, Héctor Perez, Pedro Cahn, Horacio Salomón

**Affiliations:** 1 Centro Nacional de Referencia para el SIDA, Departamento de Microbiología, Facultad de Medicina, Universidad de Buenos Aires, Capital Federal, Buenos Aires, Argentina; 2 Estación de fotobiología “Playa Unión”, Rawson, Chubut, Argentina; 3 Instituto de Virologia, Centro de Investigación en Ciencias Veterinarias y Agronómicas, INTA-Castelar, Castelar, Buenos Aires, Argentina; 4 Instituto de Medicina Tropical “Daniel A. Carrión”, Universidad Nacional Mayor San Marcos, Lima, Peru; 5 Departamento de Enfermedades Infecciosas, Hospital de Enfermedades Infecciosas “Francisco Javier Muñiz”, Capital Federal, Buenos Aires, Argentina; 6 División de Infectología, Hospital de Clínicas “José de San Martín”, Capital Federal, Buenos Aires, Argentina; 7 Servicio de Inmunocomprometidos, Hospital General de Agudos “Jose Maria Ramos Mejia”, Capital Federal, Buenos Aires, Argentina; 8 Unidad de Enfermedades Infecciosas, Hospital General de Agudos “Juan A. Fernandez”, Capital Federal, Buenos Aires, Argentina; 9 Fundación Huesped, Capital Federal, Buenos Aires, Argentina; Federal University of Sao Paulo, Brazil

## Abstract

**Background:**

Cytotoxic T-Lymphocyte (CTL) response drives the evolution of HIV-1 at a host-level by selecting HLA-restricted escape mutations. Dissecting the dynamics of these escape mutations at a population-level would help to understand how HLA-mediated selection drives the evolution of HIV-1.

**Methodology/Principal Findings:**

We undertook a study of the dynamics of HIV-1 CTL-escape mutations by analyzing through statistical approaches and phylogenetic methods the viral gene *gag* sequenced in plasma samples collected between the years 1987 and 2006 from 302 drug-naïve HIV-positive patients. By applying logistic regression models and after performing correction for multiple test, we identified 22 potential CTL-escape mutations (*p*-value<0.05; *q*-value<0.2); 10 of these associations were confirmed in samples biologically independent by a Bayesian Markov Chain Monte-Carlo method. Analyzing their prevalence back in time we found that escape mutations that are the consensus residue in samples collected after 2003 have actually significantly increased in time in one of either B or F subtype until becoming the most frequent residue, while dominating the other viral subtype. Their estimated prevalence in the viral subtype they did not dominate was lower than 30% for the majority of samples collected at the end of the 80's. In addition, when screening the entire viral region, we found that the 75% of positions significantly changing in time (p<0.05) were located within known CTL epitopes.

**Conclusions:**

Across HIV Gag protein, the rise of polymorphisms from independent origin during the last twenty years of epidemic in our setting was related to an association with an HLA allele. The fact that these mutations accumulated in one of either B or F subtypes have also dominated the other subtype shows how this selection might be causing a convergence of viral subtypes to variants which are more likely to evade the immune response of the population where they circulate.

## Introduction

HIV-1 evasion of immune response occurs through selection of mutations that impair recognition by immune effectors. Cytotoxic T-Lymphocyte response is a key factor in HIV infection by limiting viral replication, which is translated into a pronounced reduction of the viral load at the acute phase of infection [1], [2], [3]. This strong selective force leads to the rise in variants harboring CTL-escape mutations [4], [5], [6]. These mutations impair the affinity of the epitope for the HLA molecule [7], [8], [9] or the interaction between the T-cell receptor (TCR) and the peptide-HLA class I complex [10], allowing the virus to replicate more efficiently. The selection and spreading of CTL-escape mutations may impact the efficacy of immune response at a population-level, whether it is natural[11], [12] or intended to be stimulated through vaccination [13], [14], [15]. Regarding this issue, understanding the epidemiology of CTL-escape mutations is of significant interest for immunogen design and to assess how HIV might adapt to the immune response of the human population.

The aim of this study was to seek for evidence of HLA-driven evolution during the last two decades of the epidemic. Plasma samples obtained from 252 newly diagnosed individuals between the years of 1987 and 2006, together with HLA typing, were used to identify CTL-escape mutations and analyze their dynamics at a population-level. Statistical analysis used in conjunction with phylogenetic techniques allowed us to dissect the patterns of HLA-mediated evolution of HIV-1 circulating strains.

## Methods

### Subjects and Samples

All the samples included in this study were obtained from HIV newly diagnosed individuals, all of them naïve of antiretroviral drug treatment. The 103 sequences used for identification of CTL-escape mutations were obtained from samples collected at a voluntary counseling and testing site in operation at the Ramos Mejia Hospital in the city of Buenos Aires between March 2003 and April 2004. In these cases, PBMC samples were also available on which HLA typing was performed. Confidential one-on-one interviews were conducted onsite by health care workers. During these encounters, the study was explained and subjects were invited to participate. Only those subjects who were willing to participate were provided with a written informed consent, enrolled, and sampled.

The additional 149 sequences used for analysis of trends in time were retrospectively obtained from plasma samples collected between 1987 and 2006 at 5 different voluntary counseling and testing sites in the city of Buenos Aires, all of them corresponding also to antiretroviral drug naïve and newly diagnosed individuals. In these cases only the virus was characterized and no linkage to patient's data was performed. All the experiments were approved by the Research Ethics Committee of the School of Medicine, University of Buenos Aires (OHRP ref num: IORG0004063).

### Sequencing of viral RNA

Sequences were obtained by nested RT-PCR amplification of RNA extracted from plasma samples using the QIAamp viral extraction kit (QIAGEN GmbH, Hilden, Germany) and further automatic sequencing using the ABI Prism 3100/Genetic Analyzer (Applied Biosystems, CA). Sequence edition was performed using the Sequencher 4.8 software (Gene Codes Co., Ann Arbor, MI). Amplified viral regions for this study included *gag*, *pol* and *vpu* genes: HXB2 positions 682 to 2042, 2143 to 3798 and 5969 to 6595, respectively. Sequence alignment was performed by using Clustal W (BioEdit 7.0.4.1 sequence alignment editor[16]), and codon optimization was performed using GeneCutter (by Brian Gaschen, http://www.hiv.lanl.gov/content/hivdb/GENE_CUTTER/cutter.html).

For *gag* gene amplification, outer primers 1gagFW (5′-CTAGCAGTGGCGCCCGAACAGG-3′) and 1gagRev (5′-CAGTCTTTCATTTGGTGTCCTTC-3′), and inner primers 2gagFW (5′-TCTCTCGACGCAGGACTCG-3′) and 2gagRev (5′-TTTCCACATTTCCAACAGCCC-3′) were used. For *pol* gene amplification, outer primers 5CP1 (5′-GAAGGGCACACAGCCAGAAATTGCAGGG-3′) and RT3.1 (5′-GCTCCTACTATGGGTTCTTTCTCTAACTGG-3′), and inner primers 1F (5′-CAGACCAGAGCCAACAGCCCC-3′), A35 (5′-ATTGGTTGCACTTTAAATTTTCCCATTAGCCCTATT-3′), 6B (5′-CATTGTTTAACTTTTGGGCC-3′), RT3208F (5′-AACATCAGAAAGAACCTCCATT-3′), NE1 (5′-CGACCTGACAGTTACTGTATGTCTTCAATCACC-3′) and RT3798 (5′-CAAACTCCCACTCAGGAATCCA-3′) were used. For *vpu* gene amplification, outer primers PolSeq2 (5′-CGGGTTTATTACAGGGACAGC-3′) and TUE-3 (5′-TCCTTCTGCTAGACTGCCATTTA-3′), and inner primers ACC7 (5′-CTATGGCAGGAAGAAGCGGAGA-3′) and ZM140E (5′-GGGGTCAACTTTACACATGGCTTT-3′) were used. GenBank accession numbers: FJ155074 to FJ155325.

### HLA class I typing

HLA class I typing was performed on extracted genomic DNA by PCR-SSOP technique according to standard procedures.

### Statistical analysis for the identification of potential CTL-escape mutations

We performed an analysis according to the method described by Moore *et al.* [17]. First, we carried out a univariate analysis of correlations between the presence or absence of HLA alleles and the presence or absence of polymorphisms at each position of the *gag*, *pol* or *vpu* region analyzed, considering those residues that are different from the consensus sequence obtained from the same set of data as polymorphisms.

We eliminated those pairs of HLA-positions from the analysis where any of the four cells in the two-by-two table had an actual or expected count lower than 4.5. Then we performed Fisher's exact test obtaining a *p*-value that we used as a filter, eliminating those associations with a *p*-value lower than 0.1. We also estimated the power of the analysis for every HLA-position pair analyzed and eliminated that had a power lower than 30%.

We then performed a multivariate analysis by logistic regression with backwards elimination considering HLA alleles as regressors. We kept associations having a *p*-value lower than 0.05.

Then we performed a correction for multiple comparisons by calculating the *q*-value of each association by the Benjamini-Hochberg methodology. In the present study, potential CTL-escape mutations were those polymorphisms that passed through this analysis with a *q*-value lower than 0.2 (in our data set equivalent to an adjusted *p*-value lower than 0.0140).

### Correction for Multiple Comparisons

To control the inflated Type I error rate associated with multiple significance tests, the false discovery rate procedure developed by Benjamini and Hochberg was used to calculate adjusted *p*-values. This linear step-up approach controls the expected proportion of falsely rejected null hypotheses (i.e., the false discovery rate is expected to be no greater than 20%) and has some advantages (including more power) over Bonferroni-type procedures that control the family-wise error rate.

### Phylogenetic Comparative Analyses

Phylogenetic comparative analyses were performed through the BayesTraits program by Mark Pagel and Andrew Meade available at http://www.evolution.reading.ac.uk/ [18], [19]. An evolutionary model for the data was inferred using the Modeltest program[20] and the model was implemented in PAUP4.0 (http://paup.csit.fsu.edu/) to estimate branch lengths of the trees obtained as described above. For the Monte Carlo analyses, 10E6 iterations were run with a sampling frequency of 300 and a burn-in length of 50,000. For each position, the RateDev parameter was set so as to ensure a 20–40% acceptation rate. Currently there are no phylogenetic methods that take into account recombination, therefore only sequences previously characterized as “pure” (non-recombinants) could be analyzed.

### Viral Subtype Characterization

Sequences were aligned with references from the Los Alamos HIV Sequence Database (http://hiv-web.lanl.gov/content/hiv-db/SUBTYPEREF/align.html) using Clustal W (BioEdit 7.0.4.1 sequence alignment editor[16]), and codon optimization was performed using GeneCutter (by Brian Gaschen, http://www.hiv.lanl.gov/content/hivdb/GENE_CUTTER/ cutter.html) and hand aligning. After gap stripping, Neighbor-joining (NJ) trees were constructed under the Kimura two-parameter model with MEGA3.20. All sequences were individually analyzed for similarity with consensus references by SimPlot analysis (Simplot 2.5 by Stuart Ray,21 http://sray.med.som.jhmi.edu/RaySoft/SimPlot/). Sequences bearing similarities to two or more different subtypes were further analyzed by Bootscanning (Simplot 2.5 by Stuart Ray,21 http://sray.med.som.jhmi.edu/RaySoft/SimPlot/) and by visual inspection of alignments in order to identify recombination breakpoints.

### Phylogenetic Analyses

Phylogenetic trees were obtained using the TNT software (available at http://www.zmuc.dk/public/phylogeny/TNT). This software implements new technologies for dealing with large datasets [21]. We used a combination of Tree Fusing, Ratchet, Tree Drift and Sectorial Search (T-R-Df-SS) applied successively to the best trees obtained from 100 Random Addition Sequences followed by Tree Bisection Reconnection (RAS+TBR). The T-R-Df-SS was applied three times on each RAS+TBR. The best trees from each round were saved for “feeding” the subsequent rounds of T-R-Df-SS, i.e., they were fused to the trees obtained in each RAS+TBR. This scheme was repeated 10 times over 15 hours and the resulting trees were fused to verify that no further improvements of length were possible, at least under this experimental setting.

### Statistical Analyses of Trends in Time

Statistical analysis was performed by the General Linear Model Repeated Measures analysis. Samples were classified by sampling year, OR (lower or higher than 1) and viral subtype (B or F). The within-subjects factor was the sampling year with 3 levels: 1987–1997, 1998–2002 and 2003–2006. The between-subjects factors were as follows: (1) Positive associations (those with OR>1), (2) Negative associations dominating a viral subtype (those with OR<1 and the subtype where they have always been at a higher prevalence) and (3) Negative associations not dominating a viral subtype (those with OR<1 and the other subtype). The sample size for each level was 18, 4 and 4 respectively (level 1: escape at positions P28, P46, P55, P65, P81, P118, P242 and P375; level 2: escape at positions P030, P084 and P125 in subtype B and P083 in subtype F and level 3: escape at positions P030, P084 and P125 in subtype F and P083 in subtype B). Assumption of equality of covariance matrices were assessed by Box's Test (F_12; 285.5_ = 1.489; *p* = 0.127), assumption of sphericity was assessed by Mauchly's Test (*p* = 0.112), assumption of equality of error variances was assessed by Levene's Test (F_2; 21_ = 1.024; *p* = 0.376 for T1/F_2; 21_ = 0.117; *p* = 0.890 for T2/F_2; 21_ = 0.002; *p* = 0.998 for T3). Interaction between fixed factors was significant (F_4; 42_ = 13.892; p<0.001), therefore analysis for simple effects was performed by one-way ANOVA for each between-subjects level. Results for each group was F_2; 45_ = 1.543 p = 0.225 for level 1, F_2; 9_ = 1.035 p = 0.394 for level 2 and F_2; 9_ = 4,252 p = 0.050 for level 3.

## Results

### The strongest CTL response in Gag protein is mediated by HLA alleles B57 and A03

In order to compile evidence of HLA-mediated selection in the viral variants circulating in our region, we sequenced the *gag* gene in 103 samples obtained from newly diagnosed HIV-positive individuals. After sequence alignment and editing, a total of 1210-bp from the viral genome were translated into amino acids and, together with the host HLA-A and HLA-B genes characterized in the same group of patients, they were analyzed according to the methodology previously described by Moore *et al* [17] in order to identify potential CTL-escape mutations. Briefly, this analysis is based on the fact that polymorphisms that reduce the affinity of a peptide for the HLA-molecule are expected to be significantly higher in frequencies in infected individuals harboring the selective allele. Through this analysis, we found a total of 75 polymorphisms significantly associated with different HLA alleles in our population (*p*-value<0.05, logistic regression). Correcting for multiple tests, 22 out of the 75 associations had a *q*-value lower than 0.2 according to Benjamini and Hochberg method (Table 1, Figure S1). These are the viral protein sites where immune pressure is most likely to be acting. In fact, nine of them were found inside known or predicted epitopes according to Los Alamos Best-defined CTL/CD8+ Epitope Summary [22] and both the NetMHC 3.0 (CBS Prediction Server, Center for Biological Sequence Analysis, Technical University of Denmark [23]) and Epipred softwares (Microsoft Research [24]), respectively.

**Table 1:** Summary of the 22 potential CTL-escape mutations identified through statistical analysis.

	Position States					Adjusted	Epitope Analysis	
HLA	Consensus	Polimorphism	HXB2 position	OR	*p*-value	*p*-value	Sequence	Known/predicted
*Correlations strongly supported as escape by phylogenetic correction*								
A03	K	Q R	P028	21.0	<10^−7^	0.0006	RLRPGGKKK ***K***	Known
B57	T	N	P242	54.7	0.0002	0.0013	TS***T***LQEQIGWF	Known
A11	A	K M T V	P118	10.5	0.0018	0.0019	KTQQ***A***AADK	NetMHC
A03	E	D G	P055	4.8	0.0123	0.0139	-	-
*Correlations moderately supported as escape by phylogenetic correction*								
B07	G	S	P357	13.8	0.0050	0.0051	GP***G***HKARVL	Known
A24	R	K	P030	0.21	0.0076	0.0070	KY***K***LKHIVW	Known
A11	G	S	P357	13.2	0.0070	0.0063	GVGGP***G***HKAR	Known
A02	Q	H	P065	4.8	0.0031	0.0025	ILGQL***Q***PSL	Epipred/NetMHC
B40	T	A	P081	5.8	0.0084	0.0076	-	-
A24	V	L I	P046	3.4	0.0090	0.0082	-	-
*Correlations inside known or predicted epitopes but not supported by phylogenetic correction*								
A02	A	V	P083	0.13	0.0043	0.0038	SLYNTV***A***TL	Known
A01	GAP/N	S	P125	NPC	0.0105	0.0114	N***S***SQVSQNY	Epipred/NetMHC
*Correlations neither inside known or predicted epitopes, nor supported as escape by phylogenetic correction (most likely false positives)*								
B49	K	E Q A D	P012	0.00	0.0116	0.0127	-	-
B40	Q	K R N	P090	0.00	0.0104	0.0102	-	-
A02	A	P S T	P146	0.27	0.0099	0.0095	-	-
A01	L	I V M	P215	0.00	0.0105	0.0108	-	-
A31	T	S	P342	0.12	0.0063	0.0057	-	-
A31	N	S T G	P372	NPC	0.0099	0.0089	-	-
B08	T	V A	P303	4.0	0.0047	0.0044	-	-
B49	E	D	P312	15.6	0.0119	0.0133	-	-
A01	P	T S Q I	P339	4.9	0.0042	0.0032	-	-
A24	T	S	P342	5.9	0.0110	0.0121	-	-

Detailed are the positions of the mutations in HXB2 Gag protein (http://www.hiv.lanl.gov/content/sequence/ LOCATE/locate.html, see Figure S1), the HLA allele associated, the most frequent aminoacidic residue found at each position (consensus) and the polymorphisms found in those positions. All of the associations shown in the table have a *q*-value lower than 0.2 (in our data set equivalent to an adjusted *p*-value lower than 0.0140). Mutations are classified accordingly to whether they were strongly, moderately or not supported by phylogenetic correction and to whether they were or not located within known or predicted epitopes. In black-cursive are highlighted positions where escape was identified and enlarged are anchor residues for binding with the HLA molecule. In column 9 is detailed whether the mutations was located within a known epitope (“Known”) according to Los Alamos Best-defined CTL/CD8+ Epitope Summary or predicted epitope according to the NetMHC 3.0 (“NetMHC”, CBS Prediction Server, Center for Biological Sequence Analysis, Technical University of Denmark) and/or Epipred softwares (“Epipred”, Microsoft Research). NPC: not possible to calculate.

Comparative biological data might not be statistically independent due to a shared phylogenetic history and therefore violate one of the basic assumptions of most standard statistical procedures. This problem can be solved by incorporating phylogenetic information into the analyses [25]. These phylogenetic corrections help to identify false-positive results in analyses of association between HLA alleles and viral polymorphisms due to linkage relationships between individuals bearing those polymorphisms [26].Therefore, we applied this approach to our study. We first removed all the recombinant sequences from the analysis in order to avoid confounding effects on phylogeny inference. Afterwards, we applied a Bayesian Markov Chain Monte-Carlo method to the 22 associations to correct for phylogenetic correlations [19]. As shown in Table 1, we found four associations to be strongly supported after phylogenetic correction and six to be moderately supported (see also Table S1). The majority (7/10) of the associations supported by phylogenetic correction were at the same time within well-defined or predicted epitopes. Among the 12 unsupported associations, two of them were also found inside known or predicted epitopes. The remaining 10 associations were considered likely to be false-positive.

### Escapes at epitopes KYKLKHIVW and SLYNTVATL have significantly increased in time during the last twenty years of epidemic

Having identified those positions under immune pressure, we aimed to analyze the dynamics of these escape mutations at a population-level. In order to assess this issue, we first sequenced the *gag* gene from an additional set of 149 plasma samples obtained from newly diagnosed individuals from the years 1987 to 2006 (see Table S2). Then, we classified sequences according to the viral subtype and sample collection year and analyzed the frequency of those polymorphisms identified as CTL-escape mutations at three different intervals of time. Phylogenetic analysis showed that 93 (36.9%) out of the 252 samples belonged to subtype B, 55 (21.8%) were subtype F, and 103 (40.9%) were BF recombinants, which is consistent with the fact that in South America, subtype B variants co-circulate with inter-subtype BF recombinants with similar prevalence. One sample belonged to subtype C. Regardless of the high prevalence of recombinant forms between subtypes B and F, the majority of them share a common recombination pattern, which allows us to classify them according to the structures of recombination; among the 103 recombinants, 68 (66%) of them shared the same recombination pattern with a short subtype B segment of 200-bp at the 5′ of the region. In order to avoid confounding effects of recombination and to prevent the loss of analyzable samples, we eliminated those sequences that did not have the common structure and considered the remaining BF recombinants as B for those mutations located at the 5′-B segment and F for those mutations located at the 3′-F segment.

**Figure 1:**
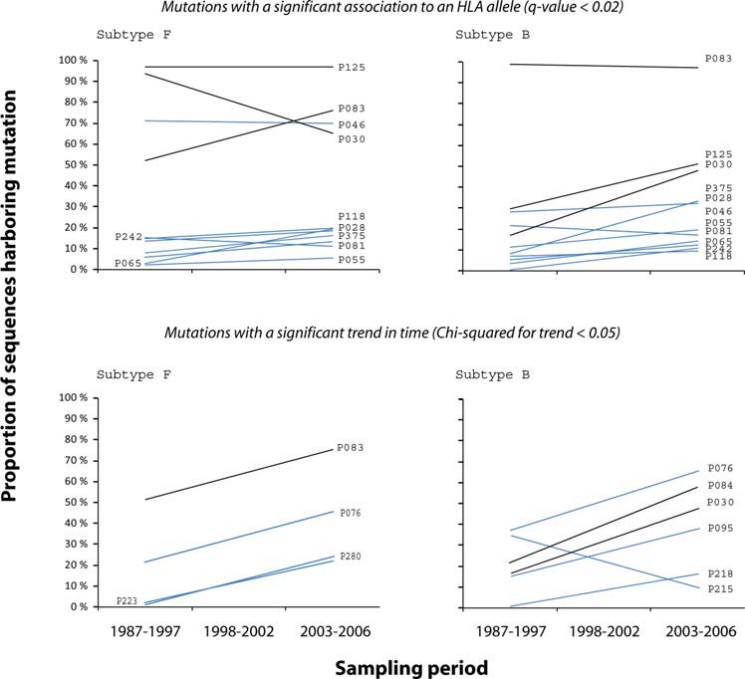
Trends in time of polymorphisms identified as potential CTL-escape mutations. Trend lines for the 3-point in time prevalence estimated are shown (prevalence estimated at each time available in table S3). Mutations were classified according to viral subtype (Subtype F in left panel, subtype B in right panel), according to whether they were identified through statistical analysis (upper panel) or trend analysis (lower panel), and according to whether they were identified through negative (black) or positive (blue) ORs. Mutations at positions P30 and P83 are repeated on upper and lower panels as they were significantly associated to an HLA allele (*q*-value<0.2) and also shown a significant trend in time (*Chi*-squared for trend <0.05). Except for mutations on positions P30 on subtype F and P215 on subtype B, trends are toward an increase or a stable prevalence in time. Particularly, those that are at present the most common state (identified through negative OR, black lines) show the higher increase in time.

As shown in Figure 1 (upper panel), we found that polymorphisms identified as escape mutations have had a wide range of behaviors. Mutations such as those identified at positions 30 and 83 have increased significantly in time (*p*<0.05, *Chi*-squared for trend) in subtype B and subtype F, respectively, while other mutations have remained either at a low prevalence as the majority, or dominating one of either subtypes B or F. Interestingly, mutations at positions 30 and 83 seem to have reached a high prevalence in time until becoming the most common residue for subtypes B and F, while they have always been at a high prevalence in subtypes F and B, respectively. The same was observed for escape at position 125, dominating subtype F and increasing in subtype B, although not statistically significant in our data set (*p* = 0.211). In fact, these three escape mutations were identified through negative associations (OR<1) as the escape is currently the consensus state at those positions. Next, we estimated site-specific synonymous (d*S*) and non-synonymous (d*N*) substitution rate parameters using a maximum-likelihood method [27], [28]: We found that 194 out of the 399 analyzed sites were subjected to a significant negative selection and 24 sites were under significant positive selection (*p*-value<0.05). Among the last group sites 76 (*p* = 0.016), 84 (*p* = 0.027), 125 (*p* = 0.007) and 280 (*p* = 0.002) were included.

### CTL selective pressures shape the evolution at a population-level of the majority of residues significantly changing in time

Next, we hypothesized that if HLA-mediated selection is a force strong enough to induce a shift in state at different viral protein positions, the spreading and accumulation of mutations that do not impair viral fitness tend to reduce the strength of the association; transmission without reversion would increase the prevalence of escape mutations in individuals not harboring the selective HLA-allele leading to a loss of statistical power [29]. Therefore, they would not be identified by statistical-based approaches. In order to test whether changes that have accumulated in other positions might have been driven by immune selection, we screened the analyzed viral region looking for other polymorphisms significantly changing in time. First, we classified samples according to sampling year and viral subtype, and then performed a *Chi*-squared analysis for trends. We found that 12 out of the total 399 positions have significantly changed over time in at least one of either the B or F subtypes (*p*-value<0.05, Table 2). Nine of them were within known CTL-epitopes and the remaining four within predicted epitopes. According to prediction software [23], nine of them are likely to impair affinity for the HLA molecule. Particularly, in the three positions where our data set had enough statistical power, we found evidence of associations with HLA alleles: two of them were already identified as potential CTL-escape mutations (Mutations in positions 30 and 83, Table 1) and mutation at position 84 had an OR of 0.34 and a *p*-value of 0.0716 for association with HLA-A02. Again, the higher changes in time were observed for those mutations identified through negative associations (Figure 1, lower panel and Table S3).

**Table 2:** Summary of the 12 polymorphisms found to have significantly changed in time during the last twenty years.

Residue position	*p*-value for trend		Position states		Sequence of known eptiopes	In our study			State that increases in time
	Subtype B	Subtype F	Consensus	Polimorphims		OR	Power	*p*-value	
***Inside known epitopes***									
P030	0.038	0.106	R	K	KW9-A24: KY***K***LKHIVW	**0.23**	**0.83**	**0.0055**	R
					GK9-B08: GGKKKY***K***LK	NC	0.42		
P076	0.029	0.030	R	K	RY11-A30: ***R***SLYNTVAVLY	1.70	0.08		K
					EV9-B08: EL ***R*** SLYNTV	1.25	0.05		
P083	0.904	0.031	A	V	SL9-A02: SLYNTV***A***TL	**0.10**	**0.94**	**0.0008**	A
					RY11-A30: RSLYNTV***A***TLY	NC	<0.01		
P084	0.012	0.730			SL9-A02: SLYNTVA***T***L	**0.34**	**0.77**	**0.0716**	V
			V	T	RY11-A30: RSLYNTVA***T***LY	1.79	0.13		
					TI9-A11: ***T***LYCVHQRI	2.83	0.30		
P095	0.088	0.040	K	R	IL10-B40: IEI***K***DTKEAL	0.82	0.15		K
P215	0.031	0.930	L	V	EL9-B40: EEAAEWDR ***L***	0.42	0.15		L
P218	0.047	0.770	V	A	AV9-B40: AEWDRLHP ***V***	2.84	0.29		A
					HA9-B07: HP***V***HAGPIA	NPC	0.03		
P223	0.123	0.009	I	V	HA9-B07: HPVHAGP***I***A	1.55	0.13		V
P280	0.601	0.020	V	T	RI8-B52: RMYSP***T***SI (1)	2.61	0.11		T
					VI9-Cw18: VRMYSP***V***SI (1)	NPC	NPC		
***Inside predicted epitopes***									
P090	0.614	0.028	Q	K R	AK9-A11: AVLYCVH***Q***K				(2)
					CK9-A03: CVH***K***KIEVK				
P054	0.555	0.009	S	A P T	EI9-B68: ET***A***EGCRQI (1)				(2)
P138	0.628	0.040	L	A F H I M P V	NL8-A24: NYPIVQN ***L***				(2)

Detailed are the *p*-values obtained by *Chi*-squared test for trend. Note that except for position P76, the other positions have significantly changed only in one of either the B or F subtype. Aminoacidic sequences of known epitopes surrounding these mutations are shown, and highlighted in black-cursive the residue that changed. Enlarged are the anchor residues for binding to the HLA molecule. Statistical results for the pair position-HLAallele in our study are detailed. Note that we only have enough statistical power to detect association at positions P30, P83 and P84. (1) In these cases no information was available of anchor residues. (2) In these cases was observed a significant increase in time of all the residues alternatives to the consensus residue as a group. NPC: not possible to calculate.

### CTL-escape mutations follow three different behaviors in time

**Figure 2:**
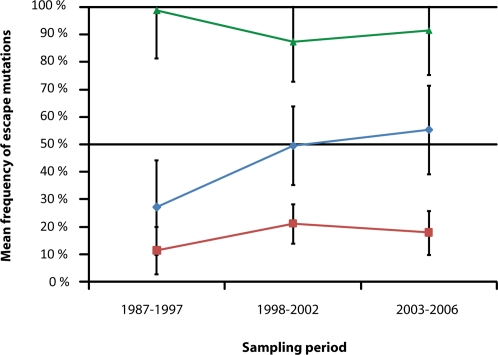
Mean frequency of escape mutations across time. Percentage of sequences harboring the escape mutations are represented on the vertical axis. As there is no previous data available to know what mutations might dominate B or F subtype, classification of mutations had to be performed *a-posteriori*: Those found to dominate a viral subtype (green line) and the remaining according to whether they were identified through negative (blue line) or positive (red line) ORs. Groups compositions were as follow: red group = P028, P046, P055, P065, P081, P118, P242 and P375 in both subtype B and F; blue group = P030, P084 and P125 in subtype B and P083 in subtype F; green group = P030, P084 and P125 in subtype F and P083 in subtype B. Statistical analysis performed by GLM Repeated Measures showed that these three groups have had significantly different behaviors in time: escape mutations identified through positive associations have always remained in a low prevalence while those identified through negative associations (as they are now the most common state) have always dominated one of either B or F subtype, or been previously in a low prevalence and increased significantly in time until becoming the most prevalent state in the subtype they do not dominate. These trends suggest a convergence of viral subtypes mediated by HLA selection.

By classifying mutations by OR it is possible to observe the different behavior in time of those mutations reaching a negative association compared with those identified through positive associations. As shown in Figure 2, the latter have always remained at a low prevalence (<30%), not changing in time (F_2, 45_ = 1.543; *p* = 0.225), while the former have significantly increased in time (F_2,9_ = 4,252; *p* = 0.050) in the subtype where they are not dominant. Interestingly, mutations found to significantly accumulate in time are associated with highly frequent HLA alleles. In fact, alleles A02 (associated with escape at positions 83 and 84), A01 (associated with escape at position 125) and A24 (associated with escape at position 30) are among the five most prevalent HLA alleles in our population according to independent data (http://www.allelefrequencies.net, Figure S2).

## Discussion

This study shows the dynamics of CTL-escape strains of HIV present in newly diagnosed individuals during the last twenty years of the epidemic. Our findings support the primary role for population-level CTL immune response in shaping the evolution of circulating HIV strains. It has been previously suggested [6], [17], [29] that negative OR (OR<1) might be the result of the accumulation of escape mutations. Particularly, Leslie *et al* [6] have previously explored this idea analyzing two common CTL-escape mutations. However, Battacharya *et al* [26] reevaluated these data and found that these highly prevalent escape mutations were more likely to have been in the founder virus of the analyzed viral subtype, supporting this conclusion by showing that the frequency of escape and non-escape forms remained constant over time. In contrast, in our study, we found four highly prevalent escape mutations and evidence to support the hypothesis that they have accumulated in time until becoming the most common state. Although only one of the four remained significantly associated with the HLA allele (Position 30 with A24) after phylogenetic correction, mutation T84V in the eighth residue of A02-restricted epitope SLYNTVATL was previously shown to diminish the specific CTL response [7]; and mutations V83A on the seventh residue of epitope variant SLYNTVVTL and S125N in the second residue of A01-restricted epitope NSSQVSQNY are predicted to diminish the affinity for the HLA molecule (IC50 104 nM to 162 nM and 61 nM to 15,205 nM, respectively [23]). The four mutations were found not to have dominated HIV variants sequenced from the early epidemic in one of either B or F subtypes, three of them showing a significant trend in time (*p*<0.05, *Chi*-squared for trend, Table 2) toward an increase in their prevalence. In fact, screening the sequenced region of *gag* for other positions significantly changing in time, we found seven out of the additional ten mutations identified through this methodology to be located within known epitopes and the remaining three within predicted epitopes. Interestingly, four of them were specifically located in anchor residues for HLA binding and three of them showed a significant association with an HLA allele. As shown in Table 2, except for those three associations, limitations in our data set preclude the analysis to detect further associations due to the lack of sufficient statistical power. Therefore, it is possible that those associations confirming an HLA-mediated selection do exist although we were not able to detect them due to the reasons stated above. Also, the lower number of sites significantly changing in time found in subtype F compared with subtype B might be related with the lower sample size for the first group and the consequent lower statistical power to detect significant trends.

Considering that at a host-level it was shown that the majority of fixed amino acids substitutions are associated with CTL-response [10], [30], our results seem to show a similar phenomenon at a population-level, suggesting that the majority of gradual increases in mutations across Gag protein might be driven by HLA-mediated immune responses.

As we analyzed HIV sequences in a time period where antiretroviral therapy was developed and became progressively more efficient, we considered the possibility that the increased prevalence we observed be driven by antiretroviral therapy mediated selection. Among Gag protein, sites under selective pressure mediated by ARV drugs would be those where Protease-mediated cleavage occurs. We found that two of the mutations analyzed (those at positions 125 and 357) were located within epitopes that overlap the two cleavage sites located within the viral region analyzed in our study: VSQNY/PIVQN and KARVL/AEAMS. However, none of the mutations analyzed in our study are located within these sites and therefore their selection is likely to be independent of those selective forces acting over these cleavage sites.

It is important to mention that we are analyzing samples at the chronic stage of infection. In fact, considering the delay from infection to diagnosis that is common in our country it is likely that the majority of samples were obtained from individuals with advanced stages of infection. It could be possible that changes in escape mutations are influenced by disease progression; however it is expected that the majority of CTL-escape mutations to be selected at early stages of infection [31] and to persist after several years of infection [10] making samples collected even at advanced stages of infection suitable for prevalence analyses of CTL-escape mutations.

By classifying samples according to OR for associations with the HLA allele, it is possible to dissect the different dynamics in time for CTL-escape mutations identified through negative or positive associations. Those identified through positive associations seem to have remained constant over time at a low frequency, while those identified through negative associations (now the consensus state) have increased significantly in time. Other mutations found to have significantly changed in time and located in HLA-binding anchor residues, such as mutations at position 76 (third residue of B08-restricted epitope ELRSLYNTV[32], [33]) in our study, might have lost their associations with the HLA-allele as a consequence of the spreading of escape variants to individuals not harboring the selective allele, even more rapidly if the selective allele is at a relatively low prevalence in the population (as is HLA-B08 for escape at position 76). The reason underlying the trend toward accumulation or not might be related with the fitness cost of the mutations selected. Among mutations identified through positive association, T242N in B57-restricted epitope TW10 is the best characterized and was shown to dramatically diminish the replication capacity of HIV [6], [34]. Also within this group is the mutation K28QR in A03-restricted epitope RK9 that was shown to be selected in the presence of A03 allele and to revert in the absence of HLA-mediated selective pressure [8], [35]. On the other hand, three out of the four mutations identified through negative associations are located in positions subjected to positive selection, therefore, able to adaptively evolve. In this sense, our analysis of dS/dN rate might shed some light on this issue as it is expected for sites that have significant relevance for viral fitness to be functionally constrained (undergoing a negative selection) while those sites that are less restricted to change may be able to evolve adaptively (undergoing positive selection). We found that while the majority of positions analyzed are subjected to negative selection (i.e. selection toward conservation of HIV genome identity), two out of the four positions (84 and 125) where escape was identified through negative ORs are subjected to positive selection. These results suggest that the accumulation of escape is possible in those positions where HIV is not highly restricted to change, while in positions undergoing negative selection, escape is more likely to revert. Also, there is evidence that mutation at position 30 (also identified through negative association) has no effect on HIV-1 replication in human T-cell lines (C.S. Adamson and E. O. Freed, personal communication).

**Figure 3:**
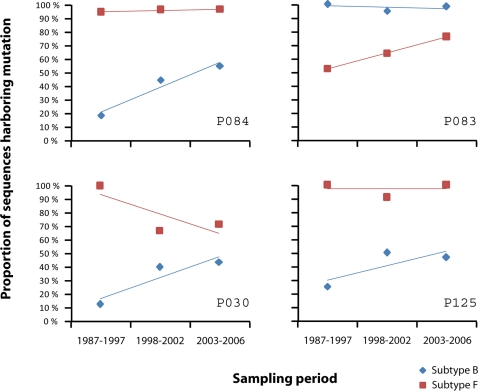
Trends in time of escape mutations identified through negative ORs in both B and F viral subtypes. The four mutations identified are currently in the majority of circulating HIV strains, regardless of viral subtype. However, analysis of their prevalence back in time showed that in the early years of epidemic they dominated only one of either the B or F subtype while they were in a low prevalence in the other subtype. This means that they have accumulated in time and driven a convergence of viral subtypes toward variants more able to evade the immune response of the human hosts.

Moreover, accumulation of these mutations in one viral subtype while dominating the other viral subtype might lead to an evolutionary convergence mediated by immune selection as shown in Figure 3 and suggests that viral subtype, at least B and F in our population, might be a relevant feature regarding adaptation to immune response as the presence of the escape in the early ancestors would give the subtype harboring it an advantage at an epitope level against immune response mediated by the corresponding HLA allele. This advantage might be extended to other viral proteins. When we repeated the statistical analysis for the identification of escape mutations over *pol* and *vpu* genes, we found that 5 out of 19 potential CTL-escape mutations were characteristic of either the B or F subtypes, again showing a potential subtype-dependent advantage for immune evasion (see Table S4).

The overall idea that viruses are adapting to more efficiently evade the human immune system across time of HIV/AIDS worldwide epidemic implies that they are increasingly more fit over time. In contrast, Arien *et al* [36] have shown that HIV viruses are increasingly less fit over time in the human host. Our results are not necessarily inconsistent with reports from Arien and colleagues as a reduced replicative fitness in head-to-head competition experiments are expected consequences for a virus that is progressively less virulent for the host; and the increased efficiency in evading the immune response at a population-level described in our study would lead to earlier rises in viral load, what in turn is expected to result in higher chances of transmission per event of exposure. Both, a lower virulence and an increased transmissibility are characteristics expected for viruses more fitted to the population of hosts where they circulate [37]. It is important to note that higher levels of viral load may lead to higher rates of CD4-positive T-cells decline which is associated with a more virulent virus; however, it has been recently shown that a decline in CD4 count and not an increase of viral load is a better predictor of disease progression [38].

In summary, we found evidence of HLA-mediated selection in HIV variants circulating in our population and the escape mutations selected to follow three different behaviors in time: remain at a low prevalence, increase significantly in time or constantly dominate an entire viral subtype. Remarkably, only one of them (mutations at position 30 in subtype F) was found to have a decreasing trend in time, although it was not significant (*p* = 0.106). In this sense our results shed some light into the rationality for vaccine design as the fact that some mutations have the potential to accumulate in time means that vaccines targeting epitopes containing them will eventually reduce their effectiveness. Therefore, to identify CTL escape mutations that are restricted to spread like those classified as “positive associations” in this study, should be a key factor for the development of future vaccines [39]. We also found that HLA-mediated selection, as it is known occurs at host-level, may also account for the majority of temporal trends of residues significantly rising in time at a population-level. Finally, the presence of the escape state as part of the natural genome of a viral subtype suggests a subtype-related evolutionary advantage and shows the force of HLA-driven evolution to induce a convergence of viral subtypes to HIV variants better able to evade the immune response of the population where they circulate.

## Supporting Information

Figure S1 Consensus sequence obtained for the HLA/polimorphism analysis. Highlighted are positions where potential CTL-escape mutationes are located, underlined those identified through statistical analyisis.
(0.97 MB PDF)

Figure S2 Phenotype frequency of HLA allele. Observed frequencies in general population (gray bars, obtained from www.allelefrequencies.net, popstudy:Argentina-Buenos Aires) and in our study (black bars) are shown. In general, frequencies observed in our HIV-1 infected population resemble the frequencies in the general population although some alleles are overrepresented (A24, A68, B39, B07, B40, A31 and B62).
(0.80 MB PDF)

Table S1 Results obtained from the phylogenetic correction analysis. Details of the results obtained from the phylogenetic correction performed over the 22 positions where polymorphisms were significantly associated with an HLA allele (q-value<0.2). Five trials were performed per position. When the best model fitted to data is the independent model, this is shown on the sixth columm.
(2.53 MB PDF)

Table S2 Details of the sequences analyzed in our study according to sampling year and viral subtype. In our study, samples were characterized as subtype B (B), F (F), C (C) or recombinants between the subtypes B and F. In this last group samples were further classified accordingly to whether they shared the same structure of recombination with one recombination spot at nucleotide 200 (989 in HXB2) in our alignment (BF200) or other recombinant structures (BF).
(1.27 MB PDF)

Table S3 Frequencies of the 18 potential CTL-escape mutations. Detail of the frequencies of the 11 polimorphisms identified as potential CTL-escape mutations through statistical analysis (mutation at position 357 was associated with two different HLA alleles) and the 7 additional identified through trend analysis, according to viral subtype.
(1.05 MB PDF)

Table S4 Results from the statistical analysis for identification of CTL-escape mutations performed over genes pol and vpu. Detailed are the p-values and ORs of the 19 sites across pol and vpu genes where evidence of CTL-escape mutations were found. Correction for multiple comparisons where performed and only associations with a q-value lower than 0.2 are shown. For those positions located within known or predicted epitopes, the aminoacidic sequence is detailed. For those significantly associated with viral subtype, p-values for association and prevalence on both B and F subtypes are provided.
(1.09 MB PDF)
