# Correction: HLA-Driven Convergence of HIV-1 Viral Subtypes B and F Toward the Adaptation to Immune Responses in Human Populations

**DOI:** 10.1371/annotation/bffe1c6a-4d1e-46bb-a8dc-648a4f566a19

**Published:** 2008-11-04

**Authors:** Dario Alberto Dilernia, Leandro Jones, Sabrina Rodriguez, Gabriela Turk, Andrea E. Rubio, Sandra Pampuro, Manuel Gomez-Carrillo, Christian T. Bautista, Gabriel Deluchi, Jorge Benetucci, María Beatriz Lasala, Leonardo Lourtau, Marcelo Horacio Losso, Héctor Perez, Pedro Cahn, Horacio Salomón

The eighth author's name was displayed incorrectly. It should be: Christian T. Bautista.

