# Supplementary material for: HLA-Driven Convergence of HIV-1 Viral Subtypes B and F Toward the Adaptation to Immune Responses in Human Populations
Source: PLoS One. 2008 Oct 21;3(10):e3429. doi: 10.1371/journal.pone.0003429 (PMC2565011; doi:10.1371/journal.pone.0003429)
Supplement: Figure S1 — Consensus sequence obtained for the HLA/polimorphism analysis. Highlighted are positions where potential CTL-escape mutationes are located, underlined those identified through statistical analyisis. (0.97 MB PDF) [file pone.0003429.s001.pdf]

....|....| ....|....| ....|....| ....|....| ....|....|
10 20 30 40 50
consensus MGARASVLSG GKLDRWEKIR LRPGGKK**K**Y**R** LKHIVWASRE LERFA**V**NPGL

....|....| ....|....| ....|....| ....|....| ....|....|
60 70 80 90 100
consensus LETS**E**GCRQI LGQL**Q**PSLQT GSEEL**R**SLYN **T**V**AV**LYCVHQ KIEV**K**DTKEA

....|....| ....|....| ....|....| ....|....| ....|....|
110 120 130 140 150
consensus LDKIEEEQNK SKQKTQQ**A**AA DKGN**S**SQVSQ NYPIVQNLQG QMVHQAISPR

....|....| ....|....| ....|....| ....|....| ....|....|
160 170 180 190 200
consensus TLNAWVKVVE EKAFSPEVIP MFSALSEGAT PQDLNTMLNT VGGHQAAMQM

....|....| ....|....| ....|....| ....|....| ....|....|
210 220 230 240 250
consensus LKDTINEEAA EWDR**L**HP**V**HA GP**I**PPGQMRE PRGSDIAGTT S**T**LQEQIQWM

....|....| ....|....| ....|....| ....|....| ....|....|
260 270 280 290 300
consensus TSNPPVPVGE IYKRWIILGL NKIVRMYSP**V** SILDIRQGPK EPFRDYVDRF

....|....| ....|....| ....|....| ....|....| ....|....|
310 320 330 340 350
consensus FKTLRAEQAT QEVKNWMTET LLVQNANPDC KTILKALGPG ATLEEMMTAC

....|....| ....|....| ....|....| ....|....| ....|....
360 370 380 390
consensus QGVGGP**G**HKA RVLAEAMSQV TNSTNVMMQK SNFKGQRKIV KCFNCGKEG

Figure S1 Consensus sequence obtained for the HLA/polimorphism analysis. Highlighted are positions where potential CTL-escape mutationes are located, underlined those identified through statistical analiyisis.
