# Supplementary material for: HLA-Driven Convergence of HIV-1 Viral Subtypes B and F Toward the Adaptation to Immune Responses in Human Populations
Source: PLoS One. 2008 Oct 21;3(10):e3429. doi: 10.1371/journal.pone.0003429 (PMC2565011; doi:10.1371/journal.pone.0003429)
Supplement: Figure S2 — Phenotype frequency of HLA allele. Observed frequencies in general population (gray bars, obtained from www.allelefrequencies.net, popstudy:Argentina-Buenos Aires) and in our study (black bars) are shown. In general, frequencies observed in our HIV-1 infected population resemble the frequencies in the general population although some alleles are overrepresented (A24, A68, B39, B07, B40, A31 and B62). (0.80 MB PDF) [file pone.0003429.s002.pdf]

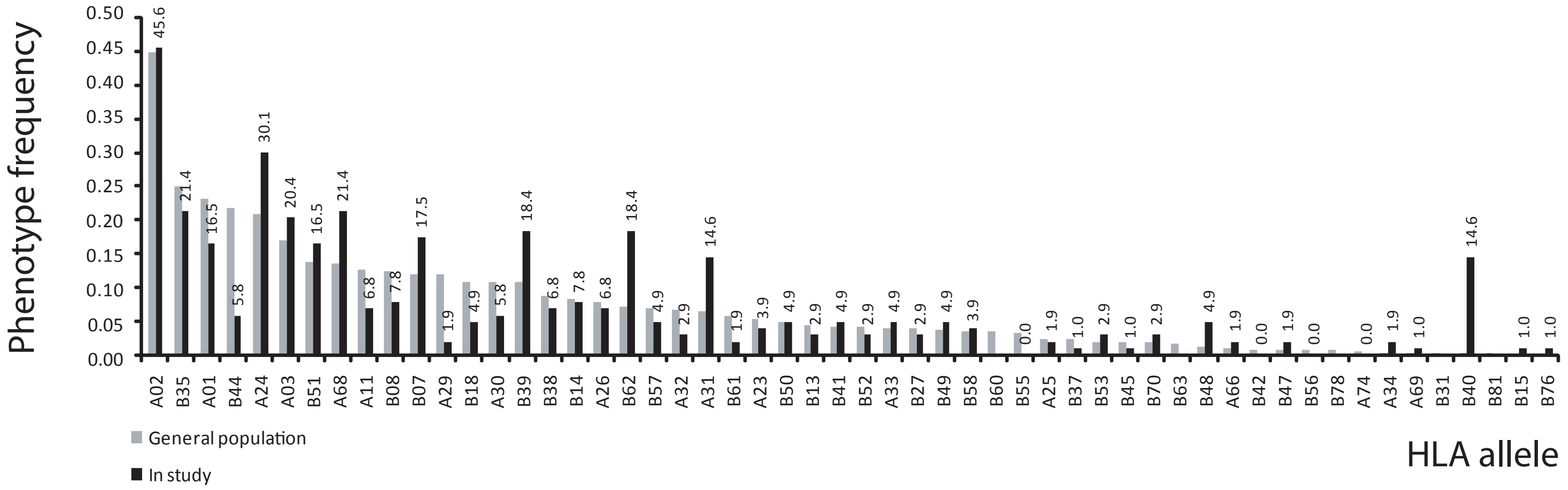

Figure S2. Phenotype frequency of HLA allele. Observed frequencies in general population (gray bars, obtained from www.allelefrequencies.net, popstudy:Argentina-Buenos Aires) and in our study (black bars) are shown. In general, frequencies observed in our HIV-1 infected population resemble the frequencies in the general population although some alleles are overrepresented (A24, A68, B39, B07, B40, A31 and B62).
