# Supplementary material for: HLA-Driven Convergence of HIV-1 Viral Subtypes B and F Toward the Adaptation to Immune Responses in Human Populations
Source: PLoS One. 2008 Oct 21;3(10):e3429. doi: 10.1371/journal.pone.0003429 (PMC2565011; doi:10.1371/journal.pone.0003429)
Supplement: Table S1 — Results obtained from the phylogenetic correction analysis. Details of the results obtained from the phylogenetic correction performed over the 22 positions where polymorphisms were significantly associated with an HLA allele (q-value<0.2). Five trials were performed per position. When the best model fitted to data is the independent model, this is shown on the sixth columm. (2.53 MB PDF) [file pone.0003429.s003.pdf]

Table S1 Results obtained from the phylogenetic correction analysis

	DEPENDENT MODEL		INDEPENDENT MODEL		
Trial	Likelihood Harmonic Mean	Acceptance rate	Likelihood Harmonic Mean	Acceptance rate	Best model or LR
**P30_A24**					
1	-79,909	-0,27	-80,979	-0,283333	2,141052
2	-80,063	-0,246667	-82,588	-0,303333	5,048832
3	-79,745	-0,256667	-80,998	-0,303333	2,505056
4	-79,869	-0,28	-80,949	-0,326667	2,160222
5	-80,034	-0,323333	-81,155	-0,316667	2,241514
**P83_A02**					
1	-74,160	-0,33	-73,432	-0,263333	Independent
2	-74,577	-0,366667	-73,703	-0,336667	Independent
3	-73,972	-0,363333	-73,675	-0,393333	Independent
4	-74,563	-0,24	-74,271	-0,33	Independent
5	-74,161	-0,313333	-73,699	-0,346667	Independent
**P125_A01**					
1	-69,711	-0,253333	-68,555	-0,216667	Independent
2	-69,651	-0,336667	-68,832	-0,25	Independent
3	-69,192	-0,233333	-69,167	-0,263333	Independent
4	-70,543	-0,333333	-68,962	-0,246667	Independent
5	-69,216	-0,29	-69,495	-0,25	0,559012
**P28_A03**					
1	-68,414	-0,313333	-77,153	-0,263333	17,477414
2	-68,686	-0,263333	-78,305	-0,26	19,23724
3	-68,630	-0,27	-77,913	-0,313333	18,564472
4	-69,576	-0,296667	-77,007	-0,226667	14,863502
5	-69,609	-0,313333	-77,644	-0,26	16,071304
**P46_A24**					
1	-74,510	-0,236667	-76,025	-0,27	3,0299
2	-74,646	-0,236667	-75,389	-0,27	1,48498
3	-74,692	-0,296667	-75,389	-0,273333	1,394476
4	-75,268	-0,37	-75,934	-0,24	1,331736
5	-74,473	-0,253333	-75,473	-0,323333	1,99874
**P81_B40**					
1	-50,939	-0,256667	-51,632	-0,33	1,38668
2	-50,381	-0,253333	-51,254	-0,343333	1,744834
3	-50,726	-0,293333	-52,302	-0,336667	3,15293
4	-50,445	-0,233333	-51,213	-0,316667	1,536788
5	-50,879	-0,316667	-51,668	-0,326667	1,577856
**P118_A11**					
1	-33,662	-0,32	-36,381	-0,323333	5,437886
2	-34,337	-0,236667	-36,532	-0,29	4,39053
3	-33,420	-0,31	-36,155	-0,326667	5,471404
4	-33,820	-0,253333	-36,029	-0,306667	4,418136
5	-33,743	-0,246667	-36,257	-0,27	5,027364
**P242_B57**					
1	-44,783	-0,246667	-48,827	-0,39	8,087374
2	-45,069	-0,36	-50,875	-0,373333	11,610946
3	-45,489	-0,303333	-49,088	-0,36	7,198572
4	-45,866	-0,296667	-49,073	-0,33	6,412982
5	-45,519	-0,356667	-49,089	-0,4	7,139418
**P303_B08**					
1	-47,024	-0,203333	-47,167	-0,223333	0,285048
2	-47,315	-0,293333	-47,264	-0,303333	Independent
3	-47,225	-0,243333	-47,288	-0,246667	0,125084
4	-47,557	-0,22	-47,498	-0,2	Independent
5	-47,926	-0,29	-47,155	-0,286667	Independent
**P65_A02**					
1	-78,613	-0,266667	-79,734	-0,336667	2,24172
2	-78,611	-0,253333	-80,122	-0,306667	3,021794
3	-78,806	-0,32	-79,944	-0,346667	2,274386
4	-78,469	-0,303333	-79,920	-0,353333	2,901506
5	-78,805	-0,343333	-79,850	-0,286667	2,090716
**P357_B07**					
1	-73,839	-0,31	-75,075	-0,373333	2,47302
2	-74,004	-0,3	-75,016	-0,353333	2,143562
3	-73,801	-0,313333	-75,054	-0,243333	2,505346
4	-73,942	-0,3	-75,784	-0,276667	3,683128
5	-73,864	-0,283333	-74,894	-0,32	2,060035

	DEPENDENT MODEL		INDEPENDENT MODEL		
Trial	Likelihood Harmonic Mean	Acceptance rate	Likelihood Harmonic Mean	Acceptance rate	Best model or LR
**P12_B49**					
1	-43,936	-0,303333	-44,618	-0,23	1,36343
2	-47,563	-0,383333	-43,912	-0,326667	Independent
3	-50,098	-0,273333	-43,184	-0,333333	Independent
4	-43,231	-0,33	-43,937	-0,29	1,411416
5	-43,256	-0,396667	-43,839	-0,356667	1,16582
**P55_A03**					
1	-58,711	-0,303333	-63,039	-0,32	8,656066
2	-58,725	-0,223333	-62,976	-0,386667	8,501514
3	-59,391	-0,28	-63,239	-0,373333	7,697352
4	-58,636	-0,243333	-63,069	-0,39	8,8667
5	-59,079	-0,363333	-63,151	-0,376667	8,144018
**P90_B40**					
1	-62,562	-0,26	-60,692	-0,283333	Independent
2	-62,627	-0,26	-60,742	-0,27	Independent
3	-61,816	-0,253333	-59,865	-0,303333	Independent
4	-62,132	-0,27	-60,111	-0,316667	Independent
5	-61,616	-0,293333	-60,263	-0,27	Independent
**P146_A02**					
1	-95,943	-0,26	-96,498	-0,353333	1,10969
2	-96,319	-0,286667	-96,548	-0,35	0,458024
3	-96,687	-0,253333	-96,542	-0,366667	Independent
4	-95,949	-0,25	-96,378	-0,313333	0,856946
5	-95,916	-0,263333	-96,524	-0,346667	1,216658
**P215_A01**					
1	-71,102	-0,31	-68,838	-0,366667	Independent
2	-69,856	-0,263333	-68,598	-0,293333	Independent
3	-69,876	-0,293333	-68,181	-0,286667	Independent
4	-69,459	-0,32	-68,119	-0,34	Independent
5	-69,365	-0,223333	-68,609	-0,343333	Independent
**P339_A01**					
1	-61,280	-0,386667	-60,927	-0,39	Independent
2	-61,818	-0,38	-60,804	-0,353333	Independent
3	-61,837	-0,4	-60,764	-0,39	Independent
4	-61,902	-0,326667	-61,042	-0,376667	Independent
5	-61,577	-0,39	-61,331	-0,343333	Independent
**P312_B49**					
1	-60,804	-0,263333	-61,359	-0,373333	1,111334
2	-62,973	-0,283333	-61,437	-0,276667	Independent
3	-60,716	-0,316667	-61,128	-0,256667	0,823558
4	-60,553	-0,306667	-61,112	-0,216667	1,119274
5	-61,315	-0,333333	-60,714	-0,226667	Independent
**P342_A24**					
1	-56,254	-0,323333	-54,463	-0,29	Independent
2	-56,548	-0,316667	-54,907	-0,296667	Independent
3	-56,623	-0,256667	-54,746	-0,316667	Independent
4	-58,037	-0,316667	-54,228	-0,306667	Independent
5	-56,630	-0,21	-54,705	-0,28	Independent
**P342_A31**					
1	-39,333	-0,326667	-38,603	-0,31	Independent
2	-39,717	-0,29	-39,587	-0,313333	Independent
3	-40,592	-0,306667	-39,134	-0,27	Independent
4	-38,993	-0,253333	-38,455	-0,223333	Independent
5	-39,465	-0,22	-38,684	-0,293333	Independent
**P357_A11**					
1	-53,572	-0,22	-55,381	-0,326667	3,318628
2	-52,822	-0,3	-54,681	-0,363333	3,71707
3	-53,117	-0,273333	-54,656	-0,333333	3,077676
4	-53,645	-0,35	-56,699	-0,31	6,109122
5	-53,122	-0,22	-56,591	-0,366667	6,938418
**P372_A31**					
1	-62,109	-0,256667	-59,910	-0,246667	Independent
2	-59,910	-0,286667	-59,792	-0,323333	Independent
3	-59,895	-0,3	-59,211	-0,223333	Independent
4	-60,503	-0,273333	-59,253	-0,236667	Independent
5	-59,829	-0,28	-59,230	-0,316667	Independent

Details of the results obtained from the phylogenetic correction performed over the 22 positions where polymorphisms were significantly associated with an HLA allele (*q*-value<0.2). Five trials were performed per position. When the best model fitted to data is the independent model, this is shown on the sixth columm.
