# Supplementary material for: HLA-Driven Convergence of HIV-1 Viral Subtypes B and F Toward the Adaptation to Immune Responses in Human Populations
Source: PLoS One. 2008 Oct 21;3(10):e3429. doi: 10.1371/journal.pone.0003429 (PMC2565011; doi:10.1371/journal.pone.0003429)
Supplement: Table S2 — Details of the sequences analyzed in our study according to sampling year and viral subtype. In our study, samples were characterized as subtype B (B), F (F), C (C) or recombinants between the subtypes B and F. In this last group samples were further classified accordingly to whether they shared the same structure of recombination with one recombination spot at nucleotide 200 (989 in HXB2) in our alignment (BF200) or other recombinant structures (BF). (1.27 MB PDF) [file pone.0003429.s004.pdf]

Table S2 Details of the sequences analyzed in our study according to sampling year and viral subtype

Sample year * Viral Subtype Crosstabulation

			Viral Subtype					
			B	BF	BF200	C	F	
Sample year	1987	Count	3	0	1	0	0	**4**
		% within Sample year	75,0%	,0%	25,0%	,0%	,0%	**100,0%**
	1988	Count	3	2	2	0	2	**9**
		% within Sample year	33,3%	22,2%	22,2%	,0%	22,2%	**100,0%**
	1989	Count	3	2	0	0	3	**8**
		% within Sample year	37,5%	25,0%	,0%	,0%	37,5%	**100,0%**
	1991	Count	3	0	0	0	0	**3**
		% within Sample year	100,0%	,0%	,0%	,0%	,0%	**100,0%**
	1992	Count	0	0	2	0	1	**3**
		% within Sample year	,0%	,0%	66,7%	,0%	33,3%	**100,0%**
	1993	Count	2	0	0	0	1	**3**
		% within Sample year	66,7%	,0%	,0%	,0%	33,3%	**100,0%**
	1994	Count	1	1	0	0	0	**2**
		% within Sample year	50,0%	50,0%	,0%	,0%	,0%	**100,0%**
	1995	Count	0	0	1	0	1	**2**
		% within Sample year	,0%	,0%	50,0%	,0%	50,0%	**100,0%**
	1996	Count	0	1	3	0	2	**6**
		% within Sample year	,0%	16,7%	50,0%	,0%	33,3%	**100,0%**
	1997	Count	1	1	1	0	1	**4**
		% within Sample year	25,0%	25,0%	25,0%	,0%	25,0%	**100,0%**
	1998	Count	3	0	3	0	3	**9**
		% within Sample year	33,3%	,0%	33,3%	,0%	33,3%	**100,0%**
	1999	Count	2	0	3	0	2	**7**
		% within Sample year	28,6%	,0%	42,9%	,0%	28,6%	**100,0%**
	2000	Count	2	5	3	0	0	**10**
		% within Sample year	20,0%	50,0%	30,0%	,0%	,0%	**100,0%**
	2001	Count	10	3	4	0	3	**20**
		% within Sample year	50,0%	15,0%	20,0%	,0%	15,0%	**100,0%**
	2002	Count	3	1	8	0	4	**16**
		% within Sample year	18,8%	6,3%	50,0%	,0%	25,0%	**100,0%**
	2003	Count	15	6	11	0	9	**41**
		% within Sample year	36,6%	14,6%	26,8%	,0%	22,0%	**100,0%**
	2004	Count	31	9	23	0	18	**81**
		% within Sample year	38,3%	11,1%	28,4%	,0%	22,2%	**100,0%**
	2005	Count	6	3	1	0	4	**14**
		% within Sample year	42,9%	21,4%	7,1%	,0%	28,6%	**100,0%**
	2006	Count	5	1	2	1	1	**10**
		% within Sample year	50,0%	10,0%	20,0%	10,0%	10,0%	**100,0%**
**Total**		**Count**	**93**	**35**	**68**	**1**	**55**	**252**
		**% within Sample yea**	**36,9%**	**13,9%**	**27,0%**	**,4%**	**21,8%**	**100,0%**

In our study, samples were characterized as subtype B (B), F (F), C (C) or recombinants between the subtypes B and F. In this last group samples were further classified accordingly to whether they shared the same structure of recombination with one recombination spot at nucleotide 200 (989 in HXB2) in our alignment (BF200) or other recombinant structures (BF).
