# Supplementary material for: HLA-Driven Convergence of HIV-1 Viral Subtypes B and F Toward the Adaptation to Immune Responses in Human Populations
Source: PLoS One. 2008 Oct 21;3(10):e3429. doi: 10.1371/journal.pone.0003429 (PMC2565011; doi:10.1371/journal.pone.0003429)
Supplement: Table S3 — Frequencies of the 18 potential CTL-escape mutations. Detail of the frequencies of the 11 polimorphisms identified as potential CTL-escape mutations through statistical analysis (mutation at position 357 was associated with two different HLA alleles) and the 7 additional identified through trend analysis, according to viral subtype. (1.05 MB PDF) [file pone.0003429.s005.pdf]

Table S3 Frequencies of the 18 potential CTL-escape mutations

Mutation	Sampling period 1987-1997	1998-2002	2003-2006	Viral subtype
Mutations with a significant association to an HLA allele (q-value < 0.02)				
**P028**	25,0%	35,0%	29,1%	B
**P028**	9,1%	25,0%	14,3%	F
**P030**	12,5%	40,0%	43,6%	B
**P030**	100,0%	66,7%	71,4%	F
**P046**	6,3%	25,0%	14,5%	B
**P046**	72,7%	66,7%	71,4%	F
**P055**	18,8%	25,0%	14,3%	B
**P055**	0,0%	8,3%	3,3%	F
**P065**	0,0%	20,0%	7,1%	B
**P065**	0,0%	16,7%	16,7%	F
**P081**	0,0%	15,0%	10,7%	B
**P081**	4,8%	12,1%	11,9%	F
**P083**	100,0%	95,0%	98,2%	B
**P083**	52,4%	63,6%	76,1%	F
**P118**	6,3%	10,0%	8,9%	B
**P118**	14,3%	18,2%	19,4%	F
**P125**	25,0%	50,0%	46,4%	B
**P125**	100,0%	90,9%	100,0%	F
**P242**	0,0%	5,0%	10,5%	B
**P242**	14,3%	15,2%	10,1%	F
**P357**	18,8%	35,0%	40,4%	B
**P357**	9,5%	18,2%	21,7%	F
Mutations with a significant trend in time (Chi-squared for trend < 0.05)				
**P076**	14,3%	45,5%	38,8%	F
**P076**	37,5%	50,0%	66,1%	B
**P084**	95,2%	97,0%	97,0%	F
**P084**	18,7%	45,0%	54,4%	B
**P095**	9,5%	3,0%	22,4%	F
**P095**	12,5%	30,0%	35,7%	B
**P215**	28,6%	18,2%	24,6%	F
**P215**	37,5%	15,0%	12,3%	B
**P218**	0,0%	18,2%	7,2%	F
**P218**	0,0%	5,0%	15,8%	B
**P223**	0,0%	12,1%	23,2%	F
**P223**	18,8%	40,0%	42,1%	B
**P280**	0,0%	12,1%	20,3%	F
**P280**	93,8%	70,0%	82,5%	B

Detail of the frequencies of the 11 polimorphisms identified as potential CTL-escape mutations through statistical analysis (mutation at position 357 was associated with two different HLA alleles) and the 7 additional identified through trend analysis, according to viral subtype.
