# Supplementary material for: HLA-Driven Convergence of HIV-1 Viral Subtypes B and F Toward the Adaptation to Immune Responses in Human Populations
Source: PLoS One. 2008 Oct 21;3(10):e3429. doi: 10.1371/journal.pone.0003429 (PMC2565011; doi:10.1371/journal.pone.0003429)
Supplement: Table S4 — Results from the statistical analysis for identification of CTL-escape mutations performed over genes pol and vpu. Detailed are the p-values and ORs of the 19 sites across pol and vpu genes where evidence of CTL-escape mutations were found. Correction for multiple comparisons where performed and only associations with a q-value lower than 0.2 are shown. For those positions located within known or predicted epitopes, the aminoacidic sequence is detailed. For those significantly associated with viral subtype, p-values for association and prevalence on both B and F subtypes are provided. (1.09 MB PDF) [file pone.0003429.s006.pdf]

Table S4 Results from the statistical analysis for identification of CTL-escape mutations performed over genes *pol* and *vpu*

				Epitope analysis		*p*-value for association	Prevalence on viral subtype	
		p-value	OR	Sequence	Known/predicted	with viral subtype	B	F
pol								
P067	B49	0,001	64,50		?	0,352		
P503	B62	0,002	12,14		?	0,513		
P273	B35	0,002	8,24	**Q**NPDIVIYQY	known	**0,013**	8,9%	100,0%
P016	B57	0,003	31,13		-	1,000		
P324	B44	0,003	28,00		-	1,000		
P310	B62	0,003	7,40		?	0,497		
vpu								
P71	B44	0,001	28,46	GE**M**GHLAPGNI / AEELAALGE**M**	CBS	0,502		
P48	B39	0,001	15,63		-	1,000		
P71	B40	0,011	4,19	GE**M**GHLAPGNI / AEELAALGE**M**	CBS	0,502		
P78	B39	0,013	0,27		-	**0,000**	88,5%	15,8%
P43	B57	0,018	11,85		-	0,180		
P70	B40	0,020	3,67	G**E**MGHLAPGNI / AEELAAL**G**EM	CBS	**0,007**	15,4%	50,0%
P3	B07	0,020	0,27	Q**P**LVILAIVAL	CBS	1,000		
P10	B44	0,021	9,88		-	0,208		
P64	B48	0,021	19,11		?	0,161		
P40	B44	0,026	8,78		-	0,180		
P2	B44	0,026	7,67		-	0,440		
P61	B39	0,029	0,33		-	**0,000**	100,0%	13,2%
P38	B44	0,033	7,80		-	**0,018**	11,3%	100,0%

Detailed are the *p*-values and ORs of the 19 sites across *pol* and *vpu* genes where evidence of CTL-escape mutations were found. Correction for multiple comparisons where performed and only associations with a *q*-value lower than 0.2 are shown. For those positions located within known or predicted epitopes, the aminoacidic sequence is detailed. For those significantly associated with viral subtype, *p*-values for association and prevalence on both B and F subtypes are provided.
